# Differential expansion of T central memory precursor and effector subsets is regulated by division speed

**DOI:** 10.1038/s41467-019-13788-w

**Published:** 2020-01-08

**Authors:** Lorenz Kretschmer, Michael Flossdorf, Jonas Mir, Yi-Li Cho, Marten Plambeck, Irina Treise, Albulena Toska, Susanne Heinzel, Matthias Schiemann, Dirk H. Busch, Veit R. Buchholz

**Affiliations:** 1 Institute for Medical Microbiology, Immunology and Hygiene, Technische Universität München (TUM), Munich, Germany; 2 German Mouse Clinic, Institute of Experimental Genetics, Helmholtz Zentrum München, Neuherberg, Germany; 3 Division of Immunology, The Walter and Eliza Hall Institute of Medical Research, Parkville, VIC Australia; 4 Department of Medical Biology, The University of Melbourne, Parkville, VIC Australia; 5 German Center for Infection Research (DZIF), Partner site Munich, Munich, Germany

**Keywords:** Cell division, Antigen presentation, Immunological memory, Cytotoxic T cells

## Abstract

While antigen-primed T cells proliferate at speeds close to the physiologic maximum of mammalian cells, T cell memory is maintained in the absence of antigen by rare cell divisions. The transition between these distinct proliferative programs has been difficult to resolve via population-based analyses. Here, we computationally reconstruct the proliferative history of single CD8^+^ T cells upon vaccination and measure the division speed of emerging T cell subsets in vivo. We find that slower cycling central memory precursors, characterized by an elongated G1 phase, segregate early from the bulk of rapidly dividing effector subsets, and further slow-down their cell cycle upon premature removal of antigenic stimuli. In contrast, curtailed availability of inflammatory stimuli selectively restrains effector T cell proliferation due to reduced receptivity for interleukin-2. In line with these findings, persistence of antigenic but not inflammatory stimuli throughout clonal expansion critically determines the later size of the memory compartment.

## Abstract (web summary)

T cell responses start with antigen-induced rapid cell divisions, and end by division cessation after pathogen clearance. Here, the authors use single-cell fate mapping and nucleoside analogue labelling to show that T central memory precursors proliferate slower than effector subsets and rely on antigenic rather than inflammatory stimuli to maintain their cell cycle speed.

## Introduction

A key feature of CD8^+^ T cell immune responses to infection or vaccination is the succession of clonal expansion, contraction and memory formation. During the contraction phase 90–95% of all T cells present at the peak of expansion die, leaving mainly memory T cells that persist long term^1,2^. Importantly, expanded T cell populations are not homogeneous but instead consist of a majority of short-lived terminal effectors (TEs), bound to die during the contraction phase, and a much smaller population of memory precursors (MPs), destined for long-term maintenance^3–6^. How this critical subset composition is regulated in parallel to the regulation of overall clonal expansion remains incompletely understood^7^. Recently, it has been elegantly shown in human yellow fever vaccinees that CD8^+^ memory T cells are derived from precursors that extensively divided during the expansion phase of the immune response^8^. Whether and how the proliferation activity of these MPs differed from that of TEs remained, however, unresolved.

In murine model systems, various lines of evidence argue that the specification of MP vs. TE fate begins early during a primary immune response, while the numerical dominance of TEs develops only toward the peak of expansion^5,9–11^. Thus, we and others have speculated that early fate decisions may coincide with the adoption of distinct and somewhat heritable proliferative behaviours that, over time, culminate in highly distinct outputs of MPs vs. TEs^12,13^. These heritable differences in proliferative behaviour could lie in the number of divisions a T cell is programmed to undergo^14–16^ or the length of time, for which it is programmed to divide before returning to quiescence^17^. Elegant in vitro studies have explored these options and found that the transcription factor c-Myc serves as an inherited timer of division activity, with T cells abruptly ceasing to divide as soon as c-Myc levels fall below a certain threshold^17^. However, this regulation of ‘division time’ has not been investigated in the context of MP vs. TE differentiation.

In fact, highly distinct outputs of MPs vs. TEs could also be programmed independently of division time—through a subset-specific regulation of ‘division speed’. Indeed, it has been shown that the speed of cell cycle progression in recently activated T cells is influenced by the type of infection or vaccination that these T cells are exposed to^18^. But again, these differences in cell cycle speed have not been resolved with respect to MP vs. TE fate. Recent work, using ex vivo live-cell imaging, suggested that MPs and TEs show similar proliferation activity throughout most of the expansion phase and start adopting distinct proliferation speeds only toward the peak of a primary immune response^19^. Instead, mathematical reconstruction of single-cell-derived immune responses in vivo argued that distinct proliferation speeds of MPs and TEs are in effect throughout the complete expansion phase and play a crucial role in establishing the characteristic subset composition of an expanded CD8^+^ T cell response^20^. Thus, it remains unclear in how far division time and/or division speed regulate the subset composition of a developing T cell immune response and when during clonal expansion this regulation sets in. In addition, it is unknown how these kinetic qualities are influenced by antigenic and inflammatory stimuli available throughout the expansion phase.

In the present study, we combined single T cell fate mapping and approaches for in vivo cell cycle analysis to directly measure the proliferation speed of MP and TE subsets emerging upon dendritic cell (DC) vaccination. We identify a proliferative hierarchy of slower cycling central memory precursors (CMPs) and more rapidly proliferating effector subsets that is fine-tuned by the sustained availability of antigenic and inflammatory stimuli throughout clonal expansion. Our results shed new light on the dynamic regulation of CD8^+^ T cell responses and could have important implications for the design of future vaccines.

## Results

### Deducing proliferation rates from single T cell fates

**Fig. 1:**
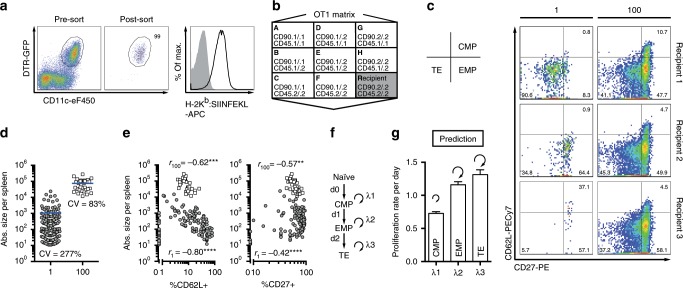
Computational modelling based on single T cell fate mapping suggests slower proliferation rates of CMPs. **a** Sorting strategy of DCs (pre-gated: CD19^−^CD3^−^) and detection of H-2K^b^ (MHC-I)-SIINFEKL after peptide-pulsing (grey: isotype control). **b** Scheme depicting genotypes of OT1 congenic matrix donors (A–H) and C57BL/6 recipients (R). **c**–**e** Progenies were recovered from spleen at day 8 after DC+*L.m*. immunization per transferred one cell (grey circles, *n* = 138) and 100 cells (white squares, *n* = 30). **c** Representative pseudo-colour plots showing expression of CD27 and CD62L for large to small single-cell-derived progenies, together with the respective 100-cell-derived progenies in the same recipients. **d** Scatter plot depicts the absolute number of daughter cells. **e** Scatter plots depict the correlation of progeny size and percentage of CD62L or CD27 expressing cells. **f** Model scheme used for fitting to the experimental data. **g** Bar graph depicts model prediction of subset-specific proliferation rates (*λ*_1_–*λ*_3_) per day, with bootstrap-error. Lines depict the mean, ‘CV' indicates the coefficient of variation (**d**). ****P < 0.0001, ***P < 0.001, **P < 0.01 (Spearman non-parametric testing (**e**)). Data are from nine independent experiments. Source data are provided as a Source Data file.

Studying the early cell cycle activity of MP and TE subsets is hampered by a lack of consensus as to when during clonal expansion these subsets fully segregate. Thus, we first utilized single T cell fate mapping and computational modelling to reconstruct the proliferative history of MP and TE subsets, based on measurements performed at the peak of the immune response. At this time point, surface markers such as CD62L and CD27 can be used to reliably identify CD62L^+^CD27^+^ CMPs, CD62L^–^CD27^+^ effector memory precursors (EMPs) and CD62L^–^CD27^–^ TEs^13,20,21^. In addition, we applied a vaccination scheme that allows to modulate the duration of antigen availability in vivo, independent of inflammatory cues^22^. Therefore, DCs expressing a diphtheria toxin receptor (DTR) transgene under control of the CD11c promoter^23^ were sorted via flow cytometry, pulsed in vitro with the SIINFEKL peptide of chicken ovalbumin (OVA) (Fig. 1a) and administered together with 2000 colony forming units (CFU) of wild-type *Listeria monocytogenes* (*L.m*.) to C57BL/6 mice. Antigenic stimuli provided by this vaccination were available in vivo at least until day 4 after DC administration (Supplementary Fig. 1). Diphtheria toxin (DTx) treatment has previously been shown to terminate these stimuli within 6–12 h of toxin injection and without a relevant influence of cross-presentation^22^.

To map the fate of individual CD8^+^ T cells responding to this ‘DC+*L.m*.’ vaccination, we adoptively transferred individual naïve T cells sorted from the peripheral blood of OT1 TCR transgenic Rag1^−/−^ donors, expressing unique combinations of the congenic markers CD45.1/.2 and CD90.1/.2 (‘OT1 matrix’; Fig. 1b). Single cells of congenic phenotypes A–G and a 100-cell control-population of congenic phenotype H were co-transferred into C57BL/6 recipients that were vaccinated 24 h later. We found that single T cell-derived expansion was highly variably (Fig. 1c, d) and that stronger expansion correlated with lower percentages of CD62L and CD27 expressing T cells per progeny (Fig. 1c, e). We have previously shown that this apparent coupling of T cell proliferation and differentiation is adequately formalized in a stochastic computational model, in which naïve CD8^+^ T cells first give rise to CMPs, which then differentiate into EMPs and TEs^20^. Applying this model structure (Fig. 1f) to the DC+*L.m*. vaccination setting, provided for an excellent model fit to both single-cell and population data (Supplementary Fig. 2a–c). Crucially, the best-fit parameters for the subset-specific proliferation and differentiation rates of this model (Supplementary Fig. 2d, e) indicated that cell cycle speed substantially increased when CMPs differentiated into their EMP and TE descendants (Fig. 1g). To refine our model predictions, we also quantified cell death of responding T cells at day 4 and 6 post immunization. While overall frequencies of dead or apoptotic cells were low at both time points, we found that non-CMPs showed on average 3.2-fold more apoptotic cells (AnnexinV^+^ FVD-eF780^–^) than CMPs (Supplementary Fig. 2f). Notably, even when introducing death rates at this ratio, CMPs were still predicted to proliferate significantly slower than EMPs and TEs (Supplementary Fig. 2g). However, this proposition stood in contrast to elegant in vitro studies, which had shown that T cells derived from the same activated progenitor undergo divisions almost simultaneously and then abruptly cease to divide^16^ at a predefined time point^17^. Thus, while the distinct cycling speeds proposed by our model served as a useful formalism, current in vitro data would suggest that activated T cells undergo concordant cell divisions and CMPs simply stop to divide earlier than their more differentiated counterparts.

### Measuring cycling activity directly in vivo

**Fig. 2:**
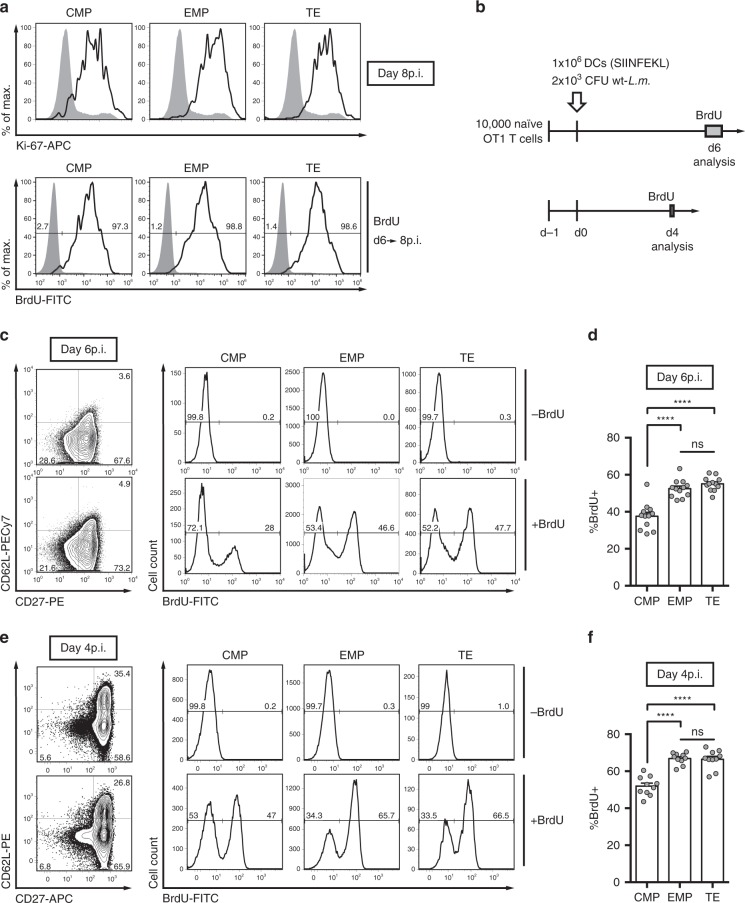
Slower cell cycle speed and not premature division cessation distinguishes CMPs from EMPs and TEs. **a** Progenies were recovered per transferred 100 naïve OT1 cells from spleen at day 8 after DC+*L.m*. immunization. Representative histograms depicting expression of cell cycle-associated Ki-67 (upper row), as well as BrdU-uptake (lower row) after repetitive 6 h pulses between day 6 and 8 p.i., for CMP, EMP and TE cells (grey: endogenous naïve CD8^+^ T cells). **b** C57BL/6 mice received 10,000 naïve OT1 cells and were immunized with DC+*L.m*. BrdU incorporation into freshly synthesized DNA was analysed at day 6 or 4 after immunization and after 16 or 3 h of labelling, respectively. **c** Representative contour plots showing the expression of CD62L and CD27 for transferred T cells, with corresponding histograms showing the BrdU profiles of the indicated subsets at day 6 (upper row: no BrdU administered). **d** Bar graph depicts the percentage of BrdU^+^ cells at day 6 (*n* = 12). **e**, **f** As in **c** and **d**, but for day 4 (*n* = 10). Naïve cells were excluded for the analysis (pre-gated: CD44^high^). Lines indicate the mean, error bars the s.e.m. ****P < 0.0001 (ANOVA). Data are representative of two independent experiments (**a**) or compiled from four (**c**, **d**) and two independent experiments (**e**, **f**). Source data are provided as a Source Data file.

With this limitation of our computational approach in mind, we set out to investigate the in vivo cycling activity of differentiating CD8^+^ T cells more directly. We adoptively transferred populations of naïve OT1 cells into hosts immunized with DC+*L.m*. (Fig. 2a) and asked, when during clonal expansion the timed cell cycle cessation, found in vitro^17^, would begin in vivo. Surprisingly, when measuring Ki-67, we found that even by day 8 post immunization all responding T cells stained positive for this marker of cell cycle activity (Fig. 2a, upper row). Since Ki-67 is fully degraded within 24–48 h after cell cycle exit^24^, this suggested that all CMPs, EMPs and TEs found at the peak of expansion had been actively proliferating at least until day 6 after immunization. To further corroborate this finding, we administered multiple pulses of bromodeoxyuridine (BrdU) between day 6 and 8 after vaccination, and found that in all T cell subsets nearly 100% of cells were BrdU^+^ after this labelling period (Fig. 2a, lower row). Therefore, virtually all CMPs, EMPs and TEs were still in cycle at day 6 post immunization. We next investigated potential differences in the cycling speed of these subsets, by administration of shorter BrdU pulses. At day 6 post immunization and after a 16-h labelling period (Fig. 2b, upper panel), we found that CMPs indeed incorporated significantly less BrdU than EMPs or TEs (Fig. 2c, d). At day 4 post immunization (Fig. 2b, lower panel), overall cycling speed appeared faster, leading to more than 60% BrdU-positive T cells within a labelling period of only 3 h. But even under these more rapid cycling conditions, CMPs incorporated significantly less BrdU than the other subsets (Fig. 2e, f). Importantly, immunization with modified vaccinia Ankara-expressing OVA (MVA-OVA), which is a potent viral vaccine vector^25^, induced a similar proliferative hierarchy (Supplementary Fig. 3).

### Measuring antigen-dependence of cycling activity in vivo

**Fig. 3:**
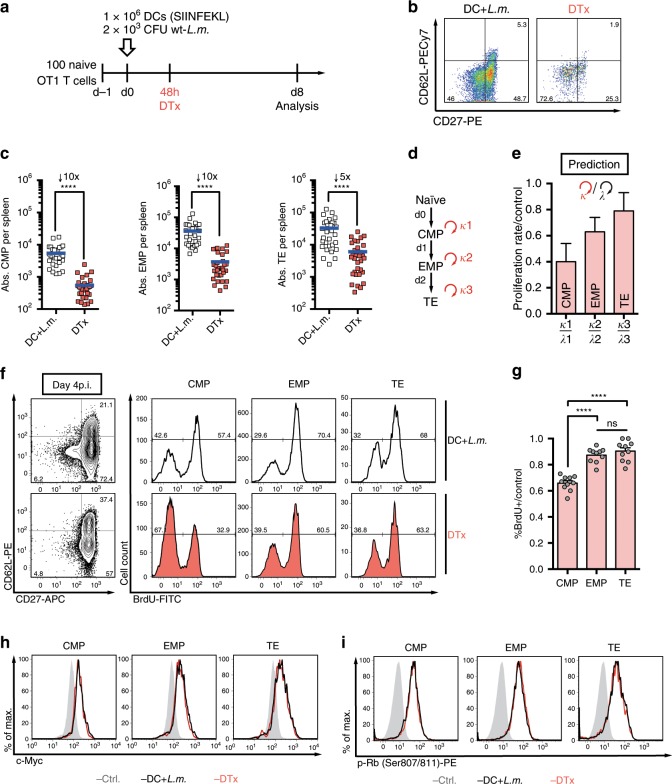
Depletion of antigenic stimuli leads to a pronounced delay in cell cycle progression of CMPs. **a** Scheme of the experimental setup and DC depletion strategy. **b**, **c** Progenies were recovered from spleen per transferred 100 OT1 cells at day 8 after immunization (*n* = 30), as well as following DTx treatment at 48 h (*n* = 32). **b** Representative pseudo-colour plots showing expression of CD62L and CD27 for transferred T cells. **c** Scatter plots depict absolute numbers of CMP, EMP and TE cells. **d** Adjusted model used for fitting to the experimental data, characterized by altered proliferation rates (*κ*_1_–*κ*_3_), effective 12 h after DTx treatment. **e** Bar graph depicts predicted proliferation rates, relative to those of the untreated control group. **f**, **g** BrdU labelling was performed as in Fig. 2e, but with DTx treatment at 48 h. **f** Representative contour plots showing the expression of CD62L and CD27 on transferred T cells, with corresponding histograms showing the BrdU profiles for the indicated subsets. **g** Bar graph depicts the percentage of BrdU^+^-cells relative to the mean of the untreated control group (*n* = 10). **h**, **i** Representative histograms show expression of (**h**) c-Myc and (**i**) retinoblastoma protein (Rb) phosphorylated at Ser807/811 (grey: endogenous naïve CD8^+^ T cells) for transferred OT1 cells at day 4 p.i. Lines indicate the mean, error bars the s.e.m.****P < 0.0001 (Mann–Whitney test). Data are compiled from four (**b**, **c**), two (**f**, **g**) or one of two independent experiments (**h**, **i**). Source data are provided as a Source Data file.

We next tested in how far the cycling speeds of CMPs, EMPs and TEs were modulated by the sustained availability of antigen. To this end, DTx was used to deplete peptide-pulsed DCs at 48 h after DC+*L.m*. immunization (Fig. 3a). When curtailing antigenic stimuli while leaving *L.m*. induced inflammation intact, absolute numbers of all responding T cell subsets were reduced. This reduction, however, was more pronounced for CMPs and EMPs (Fig. 3b) leading to a relative dominance of TEs at the peak of expansion (Fig. 3c). To answer whether this effect could be mathematically formalized by subset-specific changes in proliferation rates alone, we fitted our established model (Fig. 1f and Supplementary Fig. 2) to a dataset obtained after DC depletion, but kept differentiation rates fixed to their original values (Fig. 3d and Supplementary Fig. 4). Interestingly, the best-fit proliferation rates *kappa* obtained by this procedure remained at 80% of their DC-replete value *lambda* for TEs, but declined to 60% and 40% of that value for EMPs and CMPs, respectively (Fig. 3e). Thus, in the absence of antigenic stimuli, the cell cycle speed of CMPs was predicted to slow down relatively more than that of the other subsets. In line with this prediction, the relative reduction of BrdU incorporation upon DC depletion was found to be strongest in CMPs (Fig. 3f, g and Supplementary Fig. 5). To rule out that this effect was due to premature division cessation, we investigated c-Myc expression and phosphorylation of retinoblastoma protein (Rb) at serine residues 807/811. Both c-Myc and phosphorylated Rb are indicative of active cell cycling and, in contrast to Ki-67, are rapidly degraded and dephosphorylated, respectively, upon transition into G0 (refs. ^26,27^). We found that all responding T cell subsets maintained blastoid morphology (Supplementary Fig. 6) and stained almost uniformly positive for c-Myc (Fig. 3h) and phosphorylated Rb (Fig. 3i) under both antigen-replete and -depleted conditions. Thus, all T cell subsets remained in cycle and the observed changes in BrdU incorporation were indeed caused by a relative slowdown of cell cycle speed that was most pronounced for CMPs.

### Measuring cell cycle phase progression in vivo

**Fig. 4:**
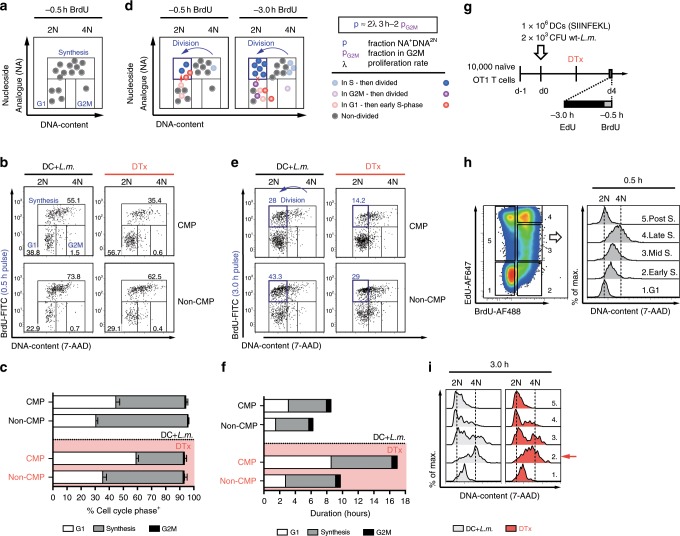
Cell cycle speed of CMPs and non-CMPs is differentially regulated by elongation of G1- and S-phase. **a** Schematic representation of cells distributed throughout all active phases of the cell cycle in NA/DNA plots. **b**, **c** Experiments were performed as in Fig. 3f, but BrdU incorporation and 7-AAD staining of DNA content were analysed 0.5 h after BrdU injection. **b** Representative dot plots showing BrdU/DNA profiles of CD62L^+^ (CMP) and CD62L^−^ (non-CMP) cells derived from transferred T cells at day 4 after immunization (DC+*L.m*.), as well as after DTx treatment (DTx). **c** Bar graph depicts the percentage of cells in the indicated cell cycle phases (DC+*L.m*. and DTx, *n* = 4). **d** Schematic representation of cell cycle progression in BrdU/ DNA plots, for cells located in different cell cycle phases at the time of NA injection, as indicated (right, bottom panel). The mathematical formula to calculate the division rate *λ* from the fraction of cells in G2M and the NA^+^DNA^2N^ gate is given (right, upper panel). **e** As in **b**, but for analysis 3.0 h after BrdU injection. **f** Bar graph depicts calculated average division times and respective cell cycle phase lengths for the indicated T cell subsets derived from transferred T cells. **g** Scheme of the experimental setup used in **h** to track S-phase progression by sequential EdU and BrdU labelling. **h** Representative pseudo-colour plot showing the EdU/BrdU-profile of transferred T cells with corresponding histograms depicting the DNA content for the indicated EdU/BrdU-subpopulations (1–5); DNA labelling 0.5 h after BrdU pulse. **i** Representative overlayed histograms showing the DNA content for the same subpopulations; DNA labelling 3.0 h after BrdU pulse. Red arrow points to slower S-phase progression upon DC depletion.  Lines indicate the mean, error bars the s.e.m. Data are compiled from two independent experiments (**b**, **c**, **e**, **f**) or are representative for one of two independent experiments (**h**, **i**). Source data are provided as a Source Data file.

We next wanted to investigate which cell cycle phases were implicated in establishing the different proliferation speeds of CMPs and non-CMPs and how their length was modulated by antigen withdrawal. A proliferating cell population’s relative distribution onto the phases of the cell cycle can be delineated by labelling active DNA synthesis through incorporation of a nucleoside analogue (NA) and in parallel measuring cellular DNA content, which ranges from diploid (2N) to tetraploid (4N). Generally, measurements of DNA content are performed approximately 0.5 h after NA administration in vitro or in vivo. This setup allows to identify the fraction of a proliferating cell population present in G1 (NA^–^2N), G2M (NA^–^4N) and S-phase (NA^+^) (Fig. 4a). We measured this distribution of cell cycle phases at day 4 post immunization for CMPs and non-CMPs under both antigen-replete and depleted conditions (Fig. 4b) and found significant subset-specific differences for distribution onto G1- and S-phase (Fig. 4c). These distributions, however, can only be translated into actual durations of cell cycle phases when the length of the overall cell cycle, and thereby the speed of its progression is known.

While elegant approaches to measure relative differences of cell cycle speed in vivo have recently been applied to B cells^28^ and hematopoietic progenitors^29^, a reliable approach for measuring absolute cell cycle speed (or length) in vivo is lacking. We developed such an approach based on the following assumptions: In theory, T cells that were in S-phase at some time during the NA-labelling period and divided before DNA content was measured, should appear as NA^+^2N (Fig. 4d, left panel, blue cells) and thereby, allow a quantification of the divided cell-fraction per time, i.e. of cell cycle speed. However, the short time frame of 0.5 h between NA administration and measurement of DNA content does not allow for sufficient separation of these divided cells from cells that have recently entered S-phase, which can also appear as NA^+^2N (Fig. 4d, left panel, red cells). To achieve this separation, we increased the time gap between administering the NA and measuring DNA content to 3 h (Fig. 4d, right panel). Given an in vivo half-life of i.p.-injected BrdU in mice of only 0.5 h^30^, this increased time gap should suffice to prevent T cells that have entered S-phase toward the tail end of the observation period from becoming NA^+^ and thus, from contaminating the NA^+^2N gate of divided cells (Fig. 4d, right panel, red and blue cells).

Indeed, we found that the fraction of BrdU^+^ T cells did not increase when analysis was performed 3 instead of 0.5 h after BrdU-pulsing (Supplementary Fig. 7a, b), arguing that no relevant label incorporation occurred beyond 0.5 h. We further confirmed the quantitative validity of our approach by a triple labelling experiment, in which the NAs ethynyldeoxyuridine (EdU) and BrdU were administered 3 and 0.5 h before measuring DNA content (Supplementary Fig. 7c): If EdU^+^ T cells exited S-phase and divided at a similar rate throughout the experiment, then a sixth of these cells should still be in S-phase at the beginning of the last 0.5 h of the 3 h observation period. In line with these assumptions, we found that approximately a sixth of all EdU^+^2N cells were also positive for BrdU (Supplementary Fig. 7d, red arrow). In order to investigate a potential influence of cell death on our measurements of NA^+^2N cells, we simulated NA/DNA experiments in silico and assumed distinct rates of cell death. This analysis showed that the percentage of NA^+^2N cells remained robust independent of varying cell death rates (Supplementary Fig. 8). Even when NA-pulsing and DNA labelling are spaced by 3 h, a small fraction of all divided T cells does not appear as NA^+^2N, since it derives from cells that had already reached G2M when the first NA pulse occurred (Fig. 4d, right panel, purple cells). We used our previously performed measurements (Fig. 4b, c) to quantify this fraction and factored it into our derivation of cell cycle speed (Fig. 4d and Supplementary Methods). Importantly, the resulting equation (Fig. 4d) requires no other parameters than this G2M fraction (p_G2M_) and the aforementioned fraction of NA^+^2N cells (p), both of which are readily accessible to direct ex vivo measurement.

When we performed such measurements at day 4 post immunization, we found that CMPs vs. non-CMPs needed an average of 8.6 vs. 6.2 h to complete their cell cycle, respectively. Strikingly, these values increased to 17.0 and 9.7 h upon depletion of antigenic stimuli (Fig. 4e, f). Thus, while both subsets slow down in the absence of antigen, cell cycle length of CMPs increased by nearly 100% and that of non-CMPs by only 50%. The distinct cell cycle lengths of CMPs and non-CMPs were mainly caused by differences in the duration of their G1 phases and a further increase of these differences upon depletion of antigenic stimuli (Fig. 4f).

Instead, depletion of antigenic stimuli led to a similar lengthening of S-phase in both CMPs and non-CMPs (Fig. 4f, DC+*L.m*. vs. DTx). To investigate whether this lengthening correlated with a delay in DNA replication, we applied a similar strategy as previously used for measuring the speed of DNA replication in germinal centre B cells^28^: We administered a first EdU and a second BrdU pulse, spaced by 2.5 h, and then measured DNA content 0.5 or 3 h after the BrdU pulse (Fig. 4g). We found that double pulsing effectively assigned T cells to G1 (EdU^–^BrdU^–^), early S (EdU^–^BrdU^+^), mid S (EdU^lo^BrdU^+^), late S/G2M (EdU^hi^BrdU^+^) and post S-phase (EdU^hi^BrdU^–^), with DNA content rising and falling from G1 (2N) to S (2–4N) to post S-phase (2N) (Fig. 4h). When delaying the measurement of DNA content from 0.5 to 3 h after NA-pulsing, we found that nearly all T cells previously identified as ‘G1’ (Fig. 4h, gate 1: EdU^–^BrdU^–^, lane 1: 2N), had increased their DNA content to >2N (Fig. 4i, lane 1) and thus had progressed towards S-phase. In accordance with slower DNA replication in the absence of antigenic stimuli, we found that T cells previously found in ‘early S-phase’ (Fig. 4h, gate 2: EdU^–^BrdU^+^, lane 2: ≥2N) had reached a higher DNA content in the antigen-replete than in the antigen-depleted setting (Fig. 4i, lane 2, red arrow).

### Investigating receptivity to inflammatory stimuli

**Fig. 5:**
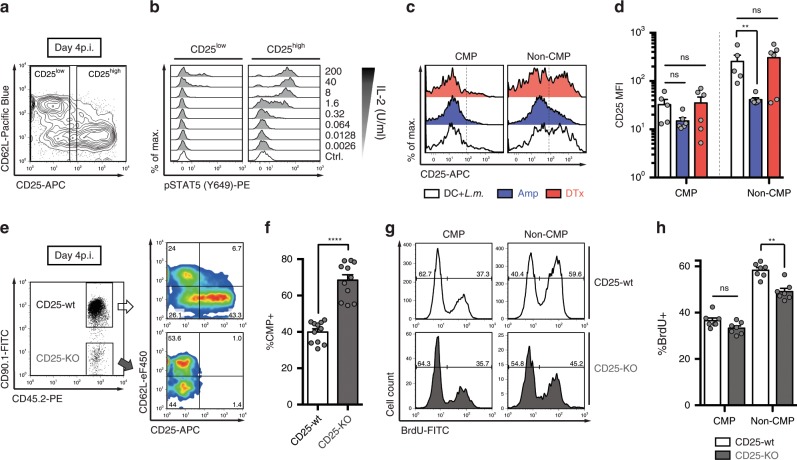
Inflammation-driven IL-2-responsiveness supports faster proliferation of non-CMPs. **a** Representative contour plot showing expression of CD62L and CD25 for progenies derived from 10^4^ naïve OT1 cells at day 4 after DC+*L.m*. immunization. **b** As in **a**, but splenocytes were stimulated ex vivo with titrated amounts of IL-2 for 15 min. Overlayed histograms showing pSTAT5 (Y649) for CD25^low^ and CD25^high^ subsets of transferred OT1 cells. **c** As in **a**, but mice were either left untreated (DC+*L.m*.; *n* = 5) or received DTx (*n* = 6) or Ampicillin (Amp; *n* = 6) treatment. Histograms showing expression of CD25 for the indicated subsets. **d** Bar graph depicts the CD25 median fluorescent intensity (MFI) values. **e**–**h** 5 × 10^4^ naïve CD25-wt (CD45.1^−/−^CD90.1^+/+^) and 5 × 10^4^ naïve CD25-KO (CD45.1^−/−^CD90.1^−/−^) OT1 cells were transferred into CD45.1^+/+^CD90.1^−/−^ recipients, followed by DC+*L.m*. immunization. BrdU was injected 3 h before analysis. **e** Representative dot plot showing the gating strategy for transferred OT1 cells (pre-gated: CD8^+^CD45.2^+^CD45.1^−^), with corresponding pseudo-colour plots showing expression of CD62L and CD25. **f** Bar graph depicts the percentage of CMP cells (*n* = 11). **g** Representative histograms showing the BrdU profiles of the indicated subsets. **h** Bar graph depicts the percentage of BrdU^+^ cells (*n* = 7). Lines depict the mean, error bars the s.e.m. **P < 0.01, ****P < 0.0001 (Mann–Whitney test). Data are from two independent experiments. Source data are provided as a Source Data file.

Having demonstrated that removal of antigenic stimuli leads to a pronounced elongation of the CMP cell cycle via G1 lengthening, we hypothesized that the more rapid transition of non-CMPs from G1- to S-phase was driven by additional TCR-independent signals. The G1/S transition in recently activated T cells can be accelerated via IL-2 signalling^31^. We found that only non-CMPs retained substantial expression of the high-affinity IL-2 receptor α-chain (CD25) at day 4 after DC+*L.m*. vaccination (Fig. 5a) and only CD25^high^ cells induced robust pSTAT5 signalling upon ex vivo IL-2 exposure (Fig. 5b). Prolonged CD25 expression can be induced by inflammatory cytokines such as IL-12 (ref. ^32^). Indeed, attenuating the inflammatory milieu during DC+*L.m*. immunization, through antibiotic treatment of *L.m*. infection, significantly reduced CD25 expression levels in non-CMPs (but not in CMPs), while DC depletion left CD25 expression levels in both subsets virtually unchanged (Fig. 5c, d). Serum levels of IL-12 and several other proinflammatory cytokines remained high when peptide-pulsed DCs were depleted in *L.m*.-infected mice, but decreased to baseline levels following Ampicillin (Amp) treatment (Supplementary Fig. 9).

To answer in how far the observed differences in CD25 expression were functionally relevant for cell cycle speed of CMPs vs. non-CMPs, we adoptively co-transferred both naïve OTI CD25 wild type (CD25-wt) and OTI CD25 knockout (CD25-KO) T cells and monitored their incorporation of BrdU at day 4 after DC+*L.m*. immunization. We found that expanding CD25-KO vs. CD25-wt populations contained a lower percentage of non-CMPs (Fig. 5e, f). More importantly, while CMPs from both CD25-wt and CD25-KO populations incorporated similar amounts of BrdU, non-CMPs from CD25-KO populations showed significantly reduced BrdU incorporation compared to their CD25-wt counterparts (Fig. 5g, h). Thus, the subset-specific and inflammation-driven expression of CD25 is at least partly responsible for the higher cell cycle speed observed in non-CMPs vs. CMPs.

### Modulating clonal expansion of memory T cells

**Fig. 6:**
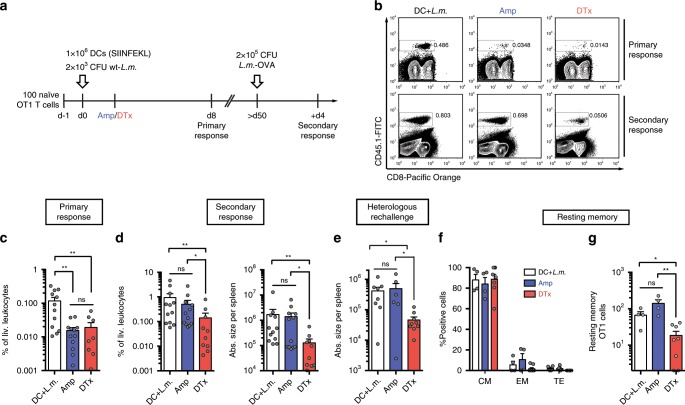
Sustained antigen availability during priming, but not inflammation supports strong memory CD8^+^ T cell responses. **a** C57BL/6 mice received 100 naïve OT1 cells and were immunized with DC+*L.m*. Mice then either received Amp at 24 h (*n* = 12), DTx at 48 h (*n* = 10), or were left untreated (DC+*L.m*.; *n* = 12). Progeny were screened in peripheral blood at day 8 and then longitudinally analysed 4 days after a rechallenge infection with recombinant *L.m*.-OVA. **b** Representative contour plots showing the percentage of transferred T cells among living leukocytes during the primary (upper row) and secondary immune response (lower row). **c** Bar graph depicts the percentage of transferred T cells among living leukocytes at day 8 (DTx: *n* = 2 progenies initially not detected). **d** Bar graphs depict the percentage of transferred T cells among living leukocytes (left) and absolute number of daughter cells in spleen (right) at day 4 after *L.m*.-OVA rechallenge. **e** As in **d**, but after heterologous MVA-OVA vaccination (DC+*L.m*. *n* = 8; Amp *n* = 6; DTx *n* = 9). **f** Bar graph indicates the percentage of resting memory T cells (no rechallenge) of central memory (CM), effector memory (EM) or TE phenotype, at 6 weeks post immunization (DC+*L.m*. *n* = 4; Amp *n* = 4; DTx *n* = 8). **g** Bar graph indicates corresponding numbers of resting memory T cells in spleen and lymph nodes. Lines depict the mean, error bars the s.e.m. *P < 0.05, **P < 0.01 (Mann–Whitney test). Data are compiled from two independent experiments. Source data are provided as a Source Data file.

In order to assess the long-term relevance of influencing division speed in emerging T cell subsets by curtailing either inflammatory or antigenic stimuli, we tracked the development of T cell memory in mice that had received Amp, DTx or no treatment in addition to DC+*L.m*. vaccination (Fig. 6a). Both interventions (DTx and Amp) similarly reduced the size of the responding T cell population at peak expansion (Fig. 6b, c). When challenging mice more than 50 days after primary immunization with a high dose of recombinant *L.m*. expressing OVA, we found that OVA-specific recall responses in the Amp-treated group reached a similar magnitude as those in the untreated vaccination group. Initial DTx treatment, however, lead to a near 10-fold reduction in magnitude of recall responses (Fig. 6b, d). Importantly, we obtained similar results when using a heterologous secondary MVA-OVA vaccination (Fig. 6e). We found that before recall immunization the vast majority of resting memory T cells in all vaccination groups displayed a CD62L^+^CD27^+^ central memory phenotype (Fig. 6f). However, absolute numbers of memory T cells that accumulated after DC+*L.m*. vaccination, as well as Amp treatment were significantly higher than those found after initial DTx treatment (Fig. 6g). Together these data further support our hypothesis that slower CMP proliferation in the absence of sustained antigenic stimuli leads to fewer CMPs at the peak of the primary response, which leads to fewer T central memory cells found in the resting memory phase and finally smaller recall responses to secondary immunization.

## Discussion

The immediate clinical relevance of this study lies in further elucidating the role of sustained antigenic and inflammatory stimuli in the generation of CD8^+^ T cell memory. Building on the results of previous work, we find that the duration of antigenic but not of inflammatory stimuli throughout the expansion phase determines the size of the memory T cell compartment^22,33–35^. These data further complete a conceptual framework, in which the size of the memory compartment is dynamically tailored to the kinetics of antigen presence during primary infection or vaccination. Meaning that, if primary infection and thereby antigen is swiftly controlled, less memory will form and—in the context of vaccination—if antigen depots quickly disperse, the magnitude of CD8^+^ T cell memory will be diminished.

The general cell biological relevance of this work lies in identifying slower cell cycle speed as a fundamental feature setting apart CMPs from their more differentiated and shorter-lived descendants. The in vivo quantification of absolute cell cycle speed in a proliferating T cell population has until now only been possible by repeat measurements of intracellular dye dilution (e.g. via CFSE). Such measurements are limited to the first few cell divisions after T cell activation. At later time points fluorescent cell cycle indicators, as present in FUCCI transgenic mice^36^, or classical NA-pulsing with DNA content measurements can determine a T cell’s position in a certain phase of the cell cycle. However, both methods cannot be used to measure cell cycle speed. To achieve this, not the static positioning but the dynamic progression of individual T cells throughout the cell cycle must be evaluated. We found that increasing the time lag between NA-pulsing and the quantification of DNA content from 0.5 to 3 h provided sufficient temporal resolution to monitor progression of individual T cells from the S- to the G1-phase.

Using this approach, we found that CMPs compared to non-CMPs showed a slower progression through the cell cycle, which was regulated, at least in part, by their lower expression of CD25. This decreased IL-2 receptivity should render CMPs more dependent on sustained antigenic stimulation for maintaining high cell cycle speeds. Indeed, premature depletion of antigenic stimuli led to pronounced cell cycle elongation in CMPs vs. non-CMPs. These data refine the classical ‘autopilot’ model of CD8^+^ T cell proliferation^37–39^, according to which a brief antigen encounter suffices to sustain CD8^+^ T cell proliferation. They argue in favour of a ‘differential autopilot’ model, in which CMPs vs. non-CMPs are less able to sustain high proliferation speeds in the absence of antigen due to their reduced capacity for sensing IL-2.

In the presence of antigen, the observed differences in subset-specific cell cycle duration may appear small (2.4 h in CMPs vs. non-CMPs). However, if overall proliferation is rapid, small differences in the average cell cycle length of distinct subsets can lead to highly distinct proliferative outputs: Transposing the cell cycle lengths observed in our study (8.6 vs. 6.2 h for CMPs vs. non-CMP in the presence of antigen) into actual proliferation rates shows that CMPs undergo an average of one division less per day (approximately 3 vs. 4 divisions per day for CMPs vs. non-CMP). Assuming that these differential proliferation activities persist for only 4 days already leads to differences in the output of CMPs vs. non-CMPs of 2^4^ - or 16-fold. Thus, already during the explosive early phase of clonal expansion, we find differences in cell cycle length of emerging T cell subsets that are sufficient to generate the highly distinct outputs of CMPs and non-CMPs found at the peak of the immune response.

Similar considerations may help to reconcile our work with literature arguing that curtailed antigen availability and earlier cessation of T cell proliferation do not diminish but enhance development of memory CD8^+^ T cells vs. shorter-lived T cell subsets^9,40^. In the face of slower cell cycle progression of CMPs vs. non-CMPs, an earlier cessation of proliferation activity, due to curtailed antigen availability^9^ or pre-existing high frequencies of memory CD8^+^ T cells^40^, should indeed generate relatively more but absolutely less CMPs. If, e.g., CMPs and non-CMPs proliferate at the above indicated speeds and do so for only 2 instead of 4 days, then the relative fraction of CMPs will increase from 1/16 (1/2^4^) to 1/4 (1/2^2^) that of non-CMPs. With 2 days less of proliferation the absolute number of CMPs will however be reduced by 64-fold (2^6^).

A previous study has argued that the cell cycle speed of recently activated T cells is modulated mainly by the length of the G1-phase^18^. The methodology provided in our work now allowed us to quantify the role of distinct cell cycle phases in modulating T cell ‘division speed’ in distinct subsets and under both antigen-replete and depleted conditions: We found that both G1 and S phases were elongated upon premature removal of antigenic stimuli. In accordance with a previous report on the cell cycle activity of germinal centre B cells, S-phase elongation was found to coincide with a slowing of DNA replication^28^. However, subset-specific differences in the cell cycle speed of CMPs vs. non-CMPs were almost exclusively due to longer G1 phases in CMPs. Thus, our data argue that TCR signalling may impact both G1- and S-phase duration while IL-2, in line with published data^31^, modulates mainly the time spent in the G1-phase.

Recently, elegant work has shown in vitro that the strength of initial T cell stimulation determines the abundance of c-Myc, which in turn regulates the time until division cessation^17^. In addition, reduced NA uptake has previously been interpreted as evidence for premature division cessation of MPs vs. TEs^9^. However, based on measurements of Ki-67 (ref. ^24^), c-Myc^17,27^ and phosphorylated Rb^26,27^, we argue that no such division cessation occurs throughout the first six days of clonal expansion. Instead, we utilized time-resolved measurements of NA uptake and DNA content to show that such differences are mainly due to distinct division speeds. Still, our data do not exclude an in vivo relevance of timed division cessation during the late phase of clonal expansion. In fact, one may argue that distinct cell cycle speeds will only translate into distinct outputs of CMPs and non-CMPs, when both subsets share the same temporally defined endpoint of proliferation. Thus, the regulation of subset-specific division speeds may be particularly relevant when division time is limited.

We show that subset-specific differences in the cell cycle speed of CMPs and non-CMPs are mainly regulated by the duration of the G1-phase. Interestingly, extremely short G1 phases of only 1–2 h, as identified for CD8^+^ T cells in our study, have been shown to facilitate the de-differentiation of somatic cells into pluripotent stem-cells, likely by constraining the time to copy epigenetic marks onto newly synthesized DNA strands^41^. Based on our data, one may hypothesize that the recently described epigenetic reprogramming during the fate specification of MPs and TEs^42^ is facilitated by an extreme shortening of the G1-phase in rapidly dividing T cells. The slower proliferation and longer G1 phases that we detected for CMPs, especially in absence of antigen, may in turn suggest that this subset returns to epigenetic stability more quickly. Indeed, recent evidence from human CD4^+^ T cells points to a proliferation-associated loss of global DNA methylation that increases from naïve to central memory to effector memory to terminally differentiated T cells^43^.

In sum, our work provides the fundamental observation that T cell fate decisions—for or against longevity—are accompanied and amplified by the adoption of distinct division speeds. By showcasing key methodology to reliably quantitate this parameter in vivo, we expect to foster further research into the intertwined mechanics of cell cycle speed and cellular differentiation, relevant for a better understanding of regulatory processes in rapidly cycling cells, within and beyond the immune system.

## Methods

### Mice and infections

Six- to 8-week-old female C57BL/6 mice were purchased from Envigo. OT-I Rag1^−/−^ matrix donor mice^21^, expressing diverse combinations of the congenic markers CD45.1/.2 and CD90.1/.2, as well as CD11c-DTR transgenic mice were bred under specific pathogen-free conditions at the mouse facility of Technische Universität München. OT1 CD25 knockout mice were bred under specific pathogen-free conditions at the Walter and Eliza Hall Institute of Medical Research animal facilities (Melbourne, Australia). Primary immunizations were performed by injecting 10^6^ SIINFEKL (OVAp)-pulsed DCs with 2 × 10^3^ CFU *Listeria monocytogenes* wild-type (*L.m*.; strain 10403s) i.v. MVA-OVA immunization was performed by injecting 10^8^ PFU i.p. For secondary infections, 2 × 10^5^ CFU of recombinant *L.m*.-OVA were administered i.v. All animal experiments were approved by the district government of upper Bavaria (Department 5—Environment, Health and Consumer Protection).

### DC isolation

DCs were isolated from CD11c-DTR transgenic donor mice, as previously described^23^. In brief, whole spleens were aseptically removed, collagenase-digested and homogenized through 100 µm cell strainers. DCs were then sorted as CD11c^+^GFP^+^ (pre-gated: live, CD3^−^CD19^−^) cells to a purity > 95%. Sorted cells were pulsed with 1 µg/ml SIINFEKL peptide (OVAp) for 1 h at 37 °C, washed twice and resuspended in PBS. Accuracy of cell numbers was confirmed by collecting a FACS-file of an appropriate aliquot directly before injection.

### DC depletion and Amp treatment

For DC depletion, diphtheria toxin (DTx; Sigma-Aldrich) was injected 48 h after DCs+*L.m*. immunization, as described^23^. Ampicillin (Amp; Carl-Roth GmbH) treatment was performed by injecting 1 mg i.p.

### Cell sorting and adoptive T cell transfer

The setup of the single-cell adoptive transfer system was previously developed in our laboratory^21,22^. Briefly, 1 or 100 naïve (CD8^+^CD44^low^) cells were successively sorted from peripheral blood of eight different OT-I Rag1^−/−^ matrix donor mice (A–H; Fig. 1b) and co-injected i.p. into C57BL/6 recipients. In some experiments, 1 × 10^4^ or 1 × 10^6^ naïve T cells were isolated from one matrix component. Cell sorting was performed on a MoFlo XDP (Beckman Coulter).

### Flow cytometry

Leukocyte suspensions from spleen- and blood samples were prepared as previously described^22^. For FACS analysis, cells were initially stained with CD16/32 (Fc-block; 93 [unlabelled]; Biolegend Cat#101301), followed by cell surface marker staining with fluorochrome-labelled antibodies specific to CD3 (145-2C11; Biolegend Cat#100308), CD8 (5H10; Cat#MCD0830), CD11c (N418; Cat#48-0114-82), CD19 (1D3; BD Biosciences Cat#562291), CD25 (PC61; Cat#17-0251-82), CD27 (LG.7F9; Cat#12-0271-83 and #17-0271-82), CD44 (IM7; Biolegend Cat#103022 and BD Biosciences Cat#562464), CD45.1 (A20; Biolegend Cat#110706, #110728 and #110722), CD45.2 (104; BD Biosciences Cat#560697 and Biolegend Cat#109808), CD62L (MEL-14; Biolegend Cat#104408, #104418, #104412 and #104424), CD69 (H1.2F3; Biolegend Cat#104505), CD90.1 (HIS51; Cat#11-0900-85 and #17-0900-82), CD90.2 (53-2.1; BD Biosciences Cat#561641), or H-2K^b^ bound to SIINFEKL (25-D1.16; Cat#17-5743-82) for 30 min at 4 °C. For Live/dead discrimination, propidium iodide (PI) or eBioscience^TM^ Fixable Viability Dye eF780 was used. Intracellular staining was performed using the eBioscience^TM^ FoxP3/Transcription Factor Staining Buffer Set, according to the manufacturer’s instructions. A fluorochrome-labelled antibody specific for Ki-67 (16A8; Biolegend Cat#652405) or a two-step staining procedure, consisting of rabbit anti-c-Myc (D84C12 [unlabelled]; Cell Signaling Cat#5605T) and Donkey anti-rabbit IgG (minimal x-reactivity; Poly4064; Biolegend Cat#406421) was used. Detection of pSTAT5 was performed after ex vivo stimulation of day 4 splenocytes with titrated amounts of IL-2 for 15 min at 37 °C, followed by immediate fixation with 2% paraformaldehyde (PFA) for 10 min at 4 °C, and subsequently 90% methanol at −20 °C overnight^5^. Fixed samples were then stained with antibodies for pSTAT5 (47/STAT5(pY694); BD Biosciences Cat#612567) and the respective cell surface antigens for 30 min at 4 °C. Detection of Retinoblastoma phosphorylated at serine residues 807/811 (clone D20B12; Cell Signaling Cat#11917S) was similarly performed, but without IL-2 restimulation. Antibodies and reagents were purchased from ThermoFisher Scientific, unless specified otherwise and were used at dilutions of 1/100–1/500. Data acquisition was performed on a CyAn ADP Lx 9-colour or CytoFLEX LX cytometer (Beckman Coulter). The number of acquired events typically ranged between 0.25 and 1 × 10^6^ for blood samples and 1 and 3 × 10^7^ for spleen samples. FACS data analysis was performed with FlowJo software (TreeStar).

### Apoptosis measurements

AnnexinV staining was performed using the FITC AnnexinV Apoptosis Detection Kit (BioLegend), according to the manufacturer’s instructions.

### Cell cycle analysis using EdU/BrdU and DNA staining

EdU and/or BrdU labelling was performed by injecting 2 mg of nucleoside analogue i.p. For the 16 h BrdU labelling time frame in Fig. 2c, d, mice were additionally maintained on 0.8 mg/ml BrdU-containing drinking water. BrdU incorporation was assessed using the eBioscience^TM^ BrdU Staining Kit for Flow Cytometry and a fluorochrome-conjugated antibody against BrdU (BU20A; Cat#11-5071-42), according to the manufacturer’s instructions. For concomitant EdU detection, the non-EdU cross-reactive anti-BrdU clone MoBU1 (Cat#MA1-12686) was used. EdU incorporation was analysed using the Click-iT^TM^ Plus EdU Flow Cytometry Assay Kit (ThermoFisher Scientific) prior to BrdU staining. Cellular DNA content was measured by staining samples with 7-Aminoactinomycin D (7-AAD; BD Biosciences) for 20 min at 4 °C immediately before acquisition. Apoptotic cells (fragmented/sub-G1 DNA content) were excluded from the analysis.

### Cytokine measurements

Serum cytokine concentrations were measured using the V-PLEX Plus Proinflammatory Panel 1 Mouse Kit (Meso Scale Discovery, Rockville, USA), according to the manufacturer’s instructions. Data acquisition and analysis was performed with the MSD instrument (Meso QuickPlex SQ120) and the included MSD discovery workbench software.

### Statistical analysis

Normality distribution of the data was assessed, using the D’Agostino & Pearson omnibus normality test. *P*-values were determined using one-way ANOVA, Mann–Whitney test and Spearman non-parametric testing (GraphPad Prism), as specified in the figure legends.

### Mathematical modelling

The stochastic modelling approach as well as implementation of cell cycle analyses are detailed in the Supplementary Information.

### Reporting summary

Further information on research design is available in the Nature Research Reporting Summary linked to this article.

## Supplementary information

Supplementary Information

Reporting Summary

## Data Availability

All relevant data included in this study can be requested from the authors. The source data underlying Figs. 1–6 and Supplementary Figs. 1–9 are provided as a Source Data file.

## Source data

Source Data

## Code availability

Mathematical codes included in this study are available upon request.
