# Supplementary material for: Differential expansion of T central memory precursor and effector subsets is regulated by division speed
Source: Nat Commun. 2020 Jan 8;11:113. doi: 10.1038/s41467-019-13788-w (PMC6949285; doi:10.1038/s41467-019-13788-w)
Supplement: Supplementary file 1 — Supplementary Information [file 41467_2019_13788_MOESM1_ESM.pdf]

Supplementary Information

(Kretschmer et al.)

Differential expansion of T central memory precursor and effector subsets is regulated by division speed

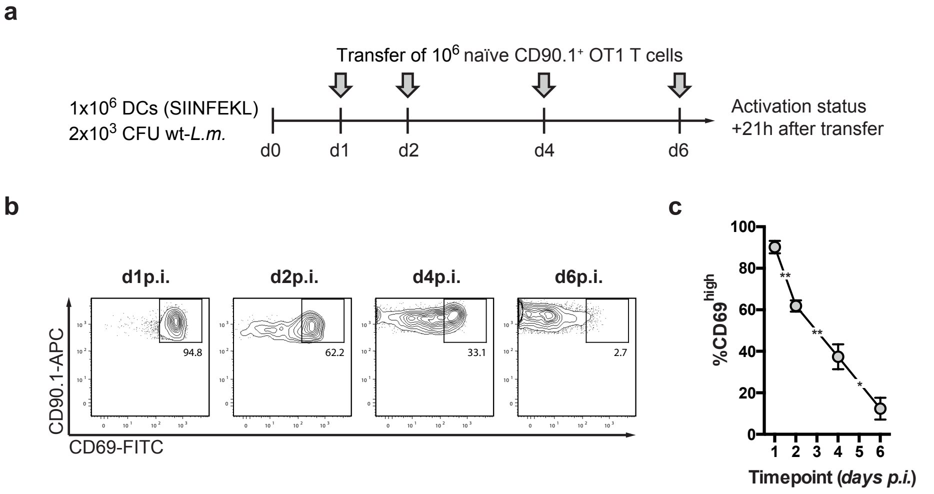

Supplementary Figure 1 | Kinetics of antigen availability after DC+*L.m.* immunization. (**a**) C57BL/6 mice were immunized using DC+*L.m.* At the indicated time points p.i., an excess number of naïve OT1 T cells were transferred and their activation status assessed 21 hours later. (**b**) Representative contour plots showing expression of CD90.1 and the early activation marker CD69 (pre-gated: $CD8^{+}CD90.1^{+}$). (**c**) Graph depicts the percentage of activated $CD69^{high}$ cells among transferred T cells (d1-4p.i. n=4; d6p.i. n=3). Symbols depict the mean, error bars the s.e.m.. $*P < 0.05$, $**P < 0.01$ (ANOVA with Tukey‘s multiple comparisons test). Data are compiled from five independent experiments. Source data are provided as a Source Data file.

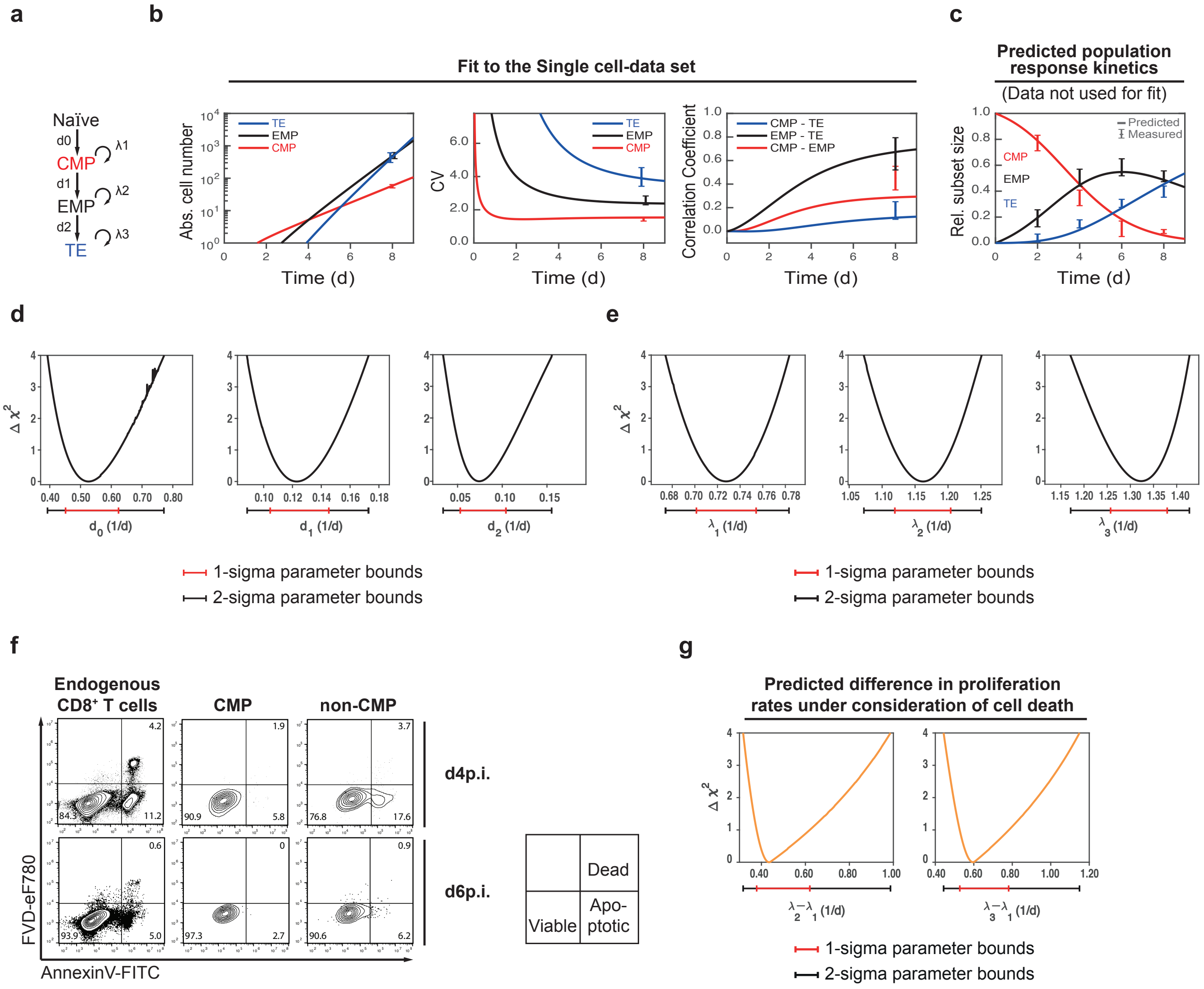

Supplementary Figure 2 | Fit of the Naïve→CMP→EMP→TE model to the single cell data set and validation of predicted population response kinetics. (**a**) Model scheme. (**b**) Best model fit to the mean absolute cell numbers, coefficients of variation (CV), and pairwise correlation coefficients for all single cell-derived subsets at day 8p.i. (**c**) Predicted response kinetics of T cell populations and experimental validation of relative subset sizes at the indicated time points after immunization. (**d-e**) Specification of all differentiation- (d_0, d_1, d_2) (**d**) and proliferation rates (λ_1, λ_2, λ_3) (**e**) with confidence intervals, assessed by the profile likelihood method. (**f**) Representative contour plots showing AnnexinV and live/dead (FVD-eF780) staining for endogenous CD8$^+$ T cells, as well as CMPs and non-CMPs of transferred OT1 cells, at day 4 and 6 after DC+*L.m.* immunization. (**g**) Specification of the predicted differences in proliferation rates between EMPs and CMPs (λ_2-λ_1), as well as TEs and CMPs (λ_3-λ_1), after introducing on average 3.2-fold higher death rates for non-CMPs, as informed by the measurements shown in (f). Source data are provided as a Source Data file.

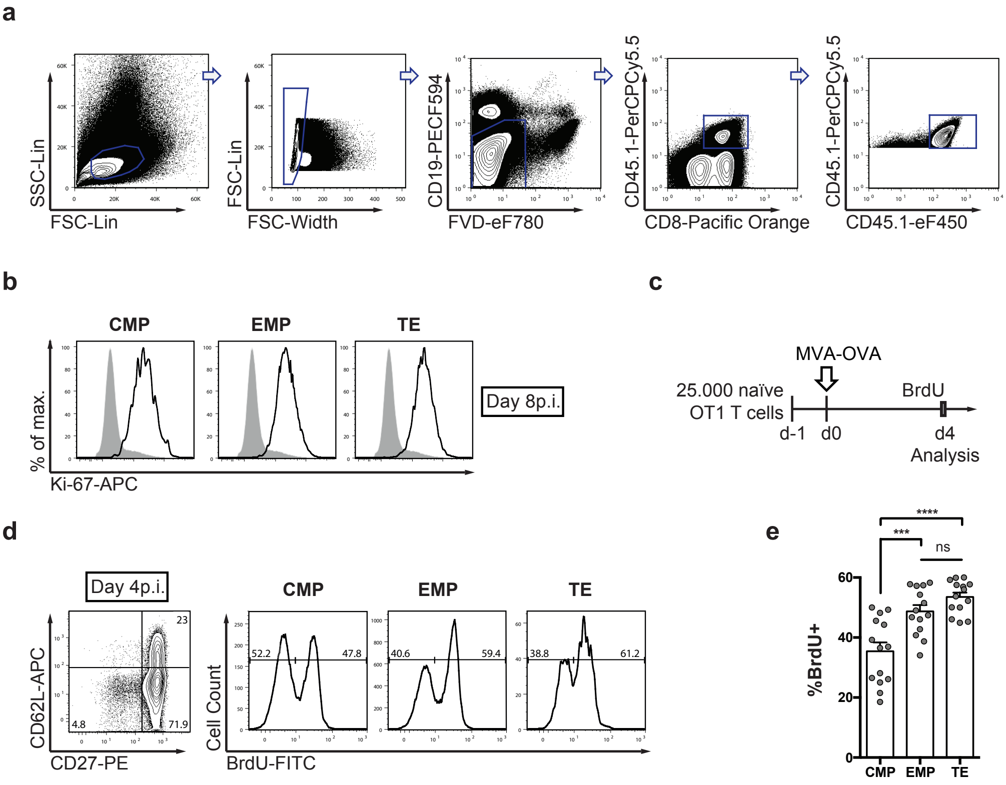

Supplementary Figure 3 | CMPs also adopt slower cell cycle speeds than EMPs and TEs during MVA-OVA vaccination. (**a**) Representative contour plots outlining the hierarchical gating strategy used to identify transferred OT1 cells (corresponding to Fig. 2). (**b**) Progenies were recovered per transferred 100 naïve OT1 cells from spleen at day 8 after MVA-OVA immunization. Representative histograms showing expression of cell cycle-associated Ki-67 for CMP, EMP, and TE cells (grey: endogenous naïve $CD8^+$ T cells). (**c**) C57BL/6 mice were transferred with $2{,}5x10^4$ naïve OT1 cells and immunized one day later with MVA-OVA. BrdU incorporation was analyzed at day 4, after 3 hours of labeling. (**d**) Representative contour plot showing the expression of CD62L and CD27 for transferred T cells, with corresponding histograms showing the BrdU-profiles for the indicated subsets. (**e**) Bar graph depicts the percentage of $BrdU^+$ cells (n=14). Lines indicate the mean, error bars the s.e.m. $^{***}P < 0.001$, $^{****}P < 0.0001$ (ANOVA). Data are from three independent experiments. Source data are provided as a Source Data file.

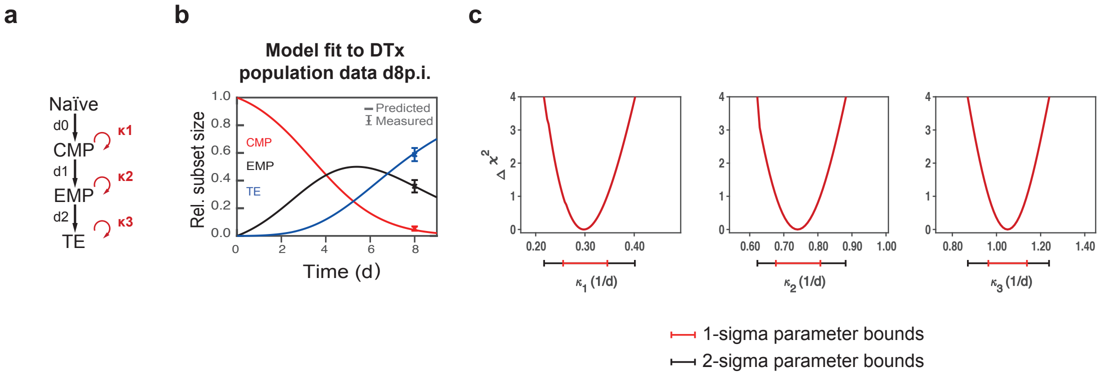

Supplementary Figure 4 | Model fit to the DC depletion data set. (**a**) Adjusted model scheme, characterized by changed proliferation rates, effective 12 hours after DTx treatment. (**b**) Fit to the d8p.i. DC depletion data set from Fig. 3 (n=32) and predicted response kinetics of T cell populations. (**c**) Specification of proliferation rates (κ_1-κ_3) with confidence intervals, assessed by the profile likelihood method. Source data are provided as a Source Data file.

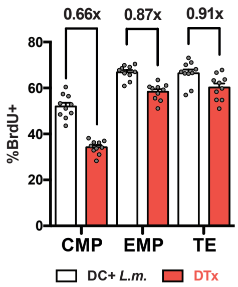

Supplementary Figure 5 | S phase transition of CMP cells is more strongly reduced than of EMP and TE cells after DC depletion. Data as in Fig. 3f-g, with fold-differences indicating the mean reduction in BrdU incorporation after DTx treatment, compared to the untreated control group (DC+*L.m.*). Lines indicate the mean, error bars the s.e.m. Source data are provided as a Source Data file.

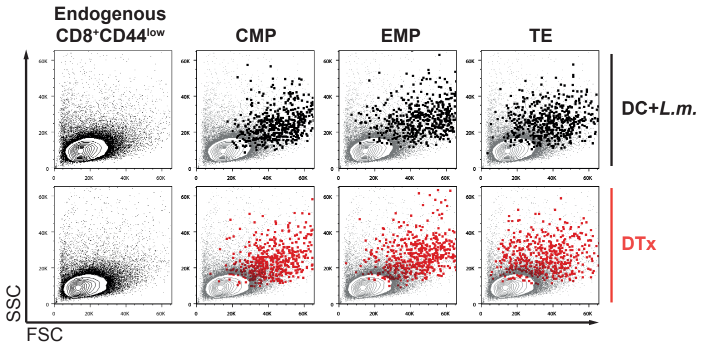

Supplementary Figure 6 | T cell blast size is not affected by DC depletion. C57BL/6 mice were transferred with 10.000 naïve OT1 cells and immunized with DC+*L.m.* Representative contour plots showing the Forward- (FSC) / Side Scatter (SSC) profiles for progenies recovered from spleen at day 4 after DC+*L.m.* immunization (black dots), as well as following DTx treatment (red dots), shown against the background of the endogenous naïve $CD8^+$ T cell compartment within the same recipients (left and grey).

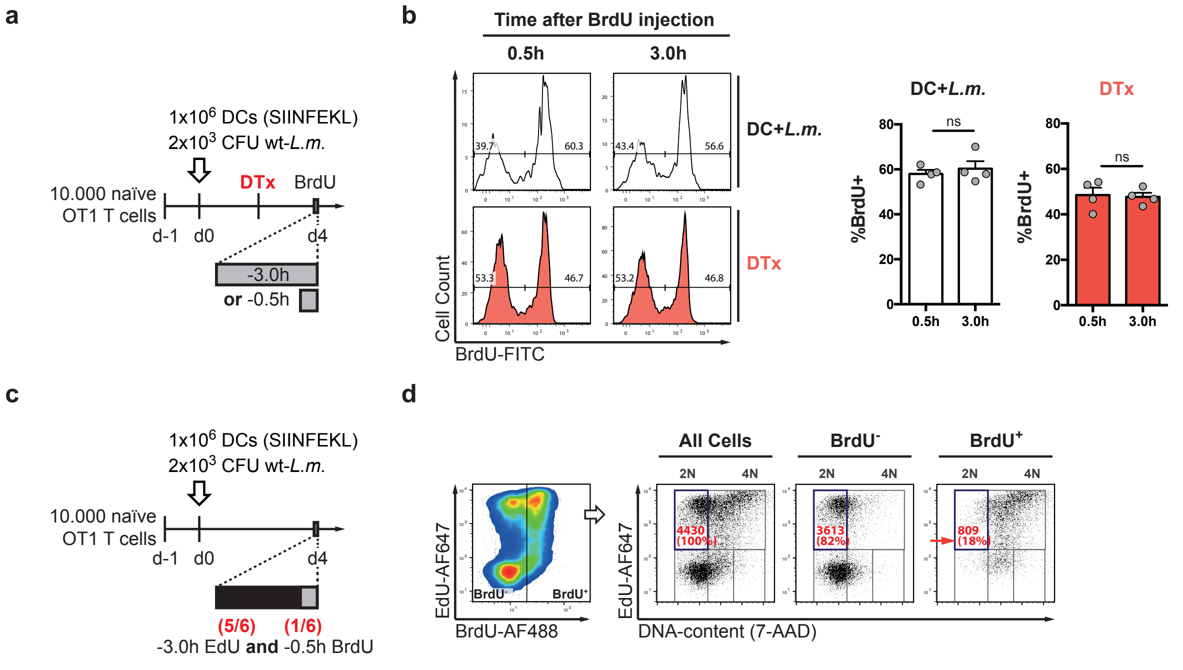

Supplementary Figure 7 | Validation of cell cycle speed measurements. (**a**) C57BL/6 mice were transferred with 10^4 naïve OT1 cells and immunized with DC+*L.m.* BrdU incorporation was analyzed at day 4 after immunization (DC+*L.m.*) as well as after DTx treatment, 0.5 or 3.0 hours after BrdU injection, respectively. (**b**) Representative histograms showing the BrdU profiles for transferred T cells with corresponding bar graphs depicting the percentage of BrdU$^+$ cells in both groups (n=4). (**c**) Scheme of the experimental setup using sequential EdU and BrdU labeling. (**d**) Representative pseudo-color plot showing the EdU/BrdU-profile for transferred T cells and corresponding dot plots showing EdU against total cellular DNA-content for all cells, BrdU$^-$, and BrdU$^+$ cells. Numbers indicate the amount of cells located in the EdU$^+$2N gate (Divided cells) and numbers in parentheses the percentage among all measured EdU$^+$2N cells (All cells). Lines indicate the mean, error bars the s.e.m. (Mann-Whitney test). Data are compiled from two independent experiments (a, b) or representative of two independent experiments (c, d). Source data are provided as a Source Data file.

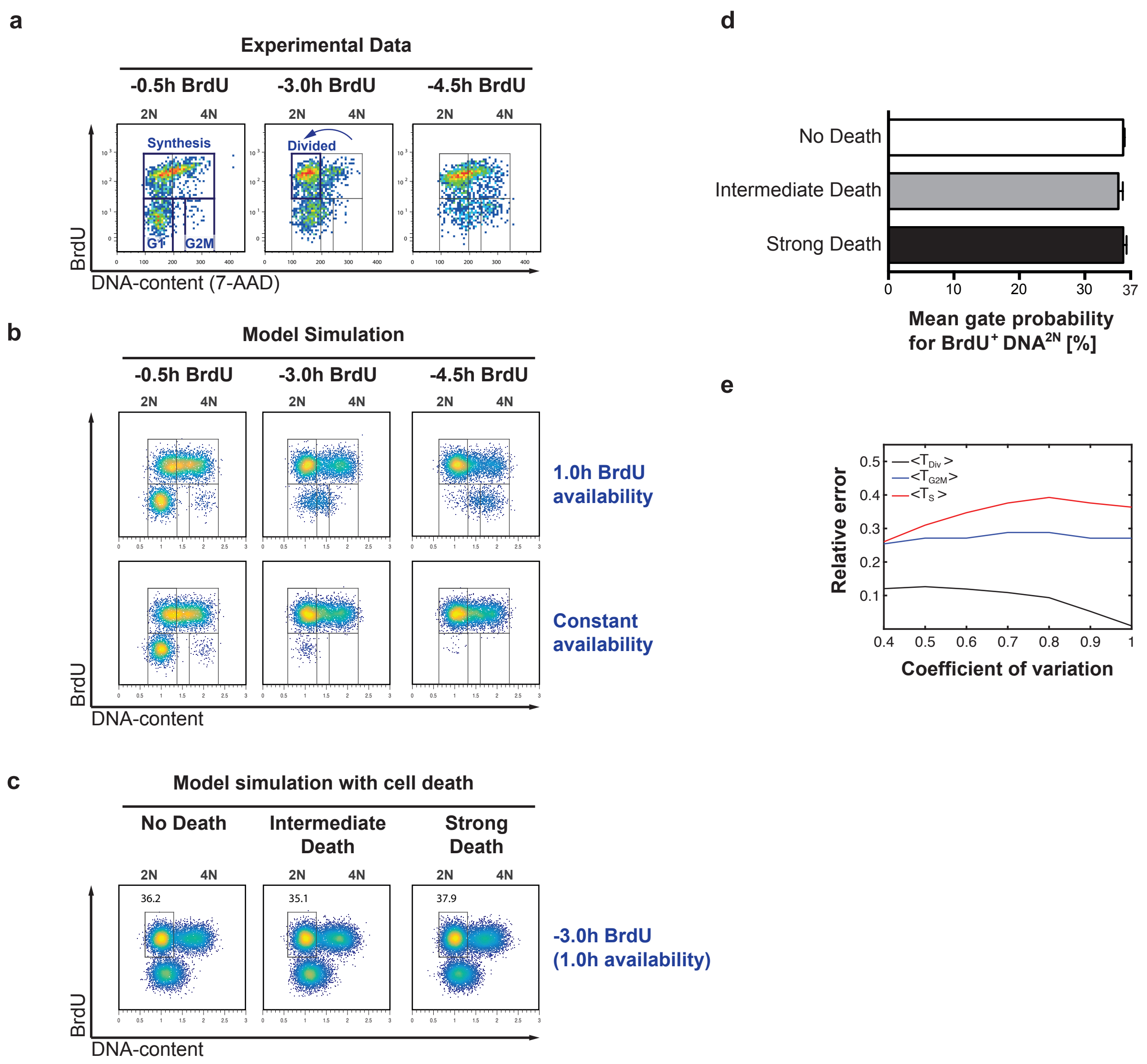

Supplementary Figure 8 | Stochastic simulation of cell cycle progression, assuming different BrdU kinetics or cell death, and small to large variabilities in the phase length distributions. (**a**) C57BL/6 mice were transferred with 50.000 naïve OT1 T cells and immunized with 5000 CFU *L.m.*-OVA. At day 4p.i. BrdU was administered at the indicated time points before analysis. Representative pseudo-colour plots showing the BrdU/DNA-profiles of transferred cells. (**b**) Cell cycle parameters were calculated based on the experimental measurements. Plots generated *in silico* showing simulated cell cycle progression, assuming 1.0 hour or constant BrdU availability, and measurement at 0.5, 3.0 or 4.5 hours after BrdU administration. (**c**) Plots show representative simulations, generated *in silico,* using interdivision times of 8.55±0.95 hours, BrdU availability of 1 hour, and an analysis time point 3.0 hours after BrdU administration. Simulations were performed in absence of cell death ("No Death"), and assuming log-normally distributed death times of 20±4 hours ("Intermediate Death") or 12±3 hours ("Strong death"). Gate is set on divided cells (BrdU+ DNA(2N)). (**d**) Bar graph indicating the probability for cells to localize in the BrdU+DNA(2N) gate, assuming no, intermediate or strong cell death. (**e**) Computed cell cycle parameters are robust with respect to variability in the phase length distributions: Based on the computed average cell cycle speed of the (CD62L^{+}) CMP population within the DC+*L.m.* group, simulated data was generated for different variabilities in the cell cycle length distribution. BrdU availability of 1 hour was assumed. The average overall cell cycle length and average phase lengths were then back calculated for a given coefficient of variation. Comparison between calculated and known parameters lead to relative errors of the mean cell cycle length (black), mean G2M length (blue), and mean S-phase length (red).

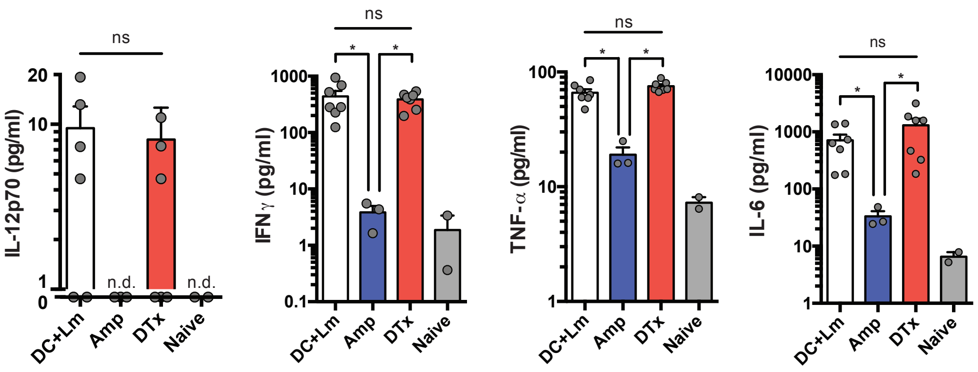

Supplementary Figure 9 | Serum concentrations of inflammatory cytokines are attenuated after Amp, but not DTx treatment. Mice were immunized and either left untreated (DC+*L.m.*; n=7) or received Amp (24h; n=3) or DTx (48h;n=7) treatment. Cytokine measurements were performed at 2.5 days after immunization. Bars depict the mean, error bars the s.e.m. *$P < 0.05$ (Mann-Whitney Test). Data are from two independent experiments. Source data are provided as a Source Data file.

Supplementary Methods

Mathematical Modeling:

Stochastic modelling approach (related to Fig. 1 and 3): All elementary model reactions (i.e., proliferation and differentiation) were assumed to be Markov processes.

The differentiation hierarchy of the model is depicted in Fig. 1g. Diphtheria toxin (DTx) treatment was modelled by resetting the proliferation rates 60 hours after immunization. Parameter estimation based on the model structure shown in Fig. 1g was done as previously described[1]. Briefly, we implemented standard least squares regression using summary statistics for the absolute cell numbers of the single-cell-derived progenies at day 8 post immunization (data shown in Fig. 1d-f) in the objective function. Our summary statistics contain mean values and coefficients of variation for all subset cell numbers, as well as Pearson correlations between the subset sizes. Non-parametric bootstrapping was used to assess the uncertainties of those quantities.

The resulting dynamics for the summary statistics of the control are shown in Supplementary Figure 2b; the corresponding parameter estimates including confidence bounds can be found in Supplementary Figure 2d, e. The 95% confidence intervals of the best-fit parameters were obtained using the profile likelihood method. The subset-specific treatment effect on proliferation was defined to be the ratio between DTx and control rate. The result of the prediction including confidence intervals is shown in Fig. 3e.

Quantification of the cell cycle (related to Fig. 4):

1. General formalism: The $\mathrm{BrdU^{+}DNA}(2N)$ gate should include descendants of cells that incorporated BrdU into the DNA, that is, whose mother cell's S-phase was overlapping with the BrdU availability window. The probability of a cell to be found in this gate at the time of measurement t_{M} is given by

$$p\left(\mathrm{BrdU^{+}DNA}(2N)\right) = \int_0^{\infty} \mathrm{d}T_{\mathrm{G2M}}\, p^{\mathrm{ss}}(T_{\mathrm{G2M}}) \int_0^{\max\,(t_{\mathrm{M}}-T_{\mathrm{G2M}},0)} \mathrm{d}a\, p^{\mathrm{ss}}\,(a)\ , \quad (1)$$

where $p^{\mathrm{ss}}(T_{\mathrm{G2M}})$ is the probability that, in steady state, the G2M length T_{G2M} (sum of the durations of the G2 and M cell cycle phases) is realized and $p^{\mathrm{ss}}(a)$ is the steady state probability of a cell having age *a.* Here the bounds of the inner integral correspond to the ages of the daughter cells at time t_{M}. If we assume that the G2M length distribution is peaked around its mean $< T_{\mathrm{G2M}} >$, then eq. (1) reduces to

$$p\big(\mathrm{BrdU^+DNA}(2N)\big) \approx \int_0^{\max\,(t_{\mathrm{M}}-<T_{\mathrm{G2M}}>,0)} \mathrm{d}a\, p^{\mathrm{ss}}\,(a). \quad (2)$$

The probability $p^{\mathrm{ss}}(a)$ is evaluated as[2]

$$p^{\mathrm{ss}}(a) = 2ce^{-ca}\int_{\mathrm{a}}^{\infty} \mathrm{d}a' D\big(T_{\mathrm{Div}} = a'\big), \quad (3)$$

where $D(T_{\mathrm{Div}})$ is the interdivision time distribution, and the exponential growth rate *c* is defined by the equation

$$2\int_0^{\infty} \mathrm{d}a\, D(T_{\mathrm{Div}} = a)\mathrm{e}^{-ca} = 1. \quad (4)$$

An approximation for the relation between *c* and $D(T_{\mathrm{Div}})$ is given by

$$c \approx \frac{\ln(2)}{<D(T_{\mathrm{Div}})>}; \quad (5)$$

this approximation is only exact for non-varying interdivision times. However, we checked the robustness of the approximations in equations (2) and (5) towards interdivision time distributions with considerable variability using stochastic simulations (Supplementary Figure 8e).

If a minimum interdivision time $T_{\mathrm{Div}}^{\mathrm{Min}}$ exists, such that $D(T_{\mathrm{Div}} < T_{\mathrm{Div}}^{\mathrm{Min}}) = 0$, then eq. (3) for ages $a < T_{\mathrm{Div}}^{\mathrm{Min}}$ reduces to $p^{\mathrm{ss}}(a) = 2ce^{-ca}$. Hence, the probability of a cell to have an age within the interval $a_1 < a < a_2$, for $a_1 < a_2 < T_{\mathrm{Div}}^{\mathrm{Min}}$, then reads

$$p^{\mathrm{ss}}(a_1 < a < a_2) = \int_{a_1}^{a_2} \mathrm{d}a\, p^{\mathrm{ss}}\,(a) = 2(e^{-ca_1} - e^{-ca_2}). \quad (6)$$

If further approximation (5) holds, evaluation of eq. (6) for the age interval describing the divided and labelled cells, gives

$$p(\mathrm{BrdU^+DNA}(2N)) = 2\big(1 - e^{-c(t_{\mathrm{M}}-<T_{\mathrm{G2M}}>)}\big). \quad (7)$$

For small exponents, $c\,(t_{\mathrm{M}} -< T_{\mathrm{G2M}} >) << 1$, eq. (7) reduces to the approximation

$$p\big(\mathrm{BrdU^+DNA}(2N)\big) \approx 2ct_{\mathrm{M}} - 2c < T_{\mathrm{G2M}} >. \quad (8)$$

Alternatively, one can switch to a description in terms of $\delta = T_{\mathrm{Div}} - a$, i.e., the cell's remaining time to divide as it was put forward in[3]. Our analogue of eq. (6) then becomes

$$p_{\mathrm{tnd}}^{\mathrm{ss}}(\delta_1 < \delta < \delta_2) = e^{c\delta_2} - e^{c\delta_1}, \quad (9)$$

where $p_{\mathrm{tnd}}^{\mathrm{ss}}(\delta)$ gives the steady state probability of a cell having a time to next division (tnd) of exactly δ left.

The probability of a cell to have a δ that lies within the interval [0;$< T_{\mathrm{G2M}} >$] is, by means of eq. (9), given by $e^{c<T_{\mathrm{G2M}}>} - 1 \approx c < T_{\mathrm{G2M}} >$. Hence, the last term in eq. (7) can be identified with the fraction of cells that are either in the G2- or M-phases of their cell cycle, resulting in

$$p\left(\mathrm{BrdU}^{+}\mathrm{DNA}(2N)\right) \approx 2ct_{\mathrm{M}} - 2p_{\mathrm{G2M}}. \quad (10)$$

This approximation has a straight forward interpretation: Our gate of interest receives contributions from all cells that divide during the experiment (first term of eq. (10)) except of those that were in the G2 or M phase at the beginning of BrdU labeling (last term of eq. (10)). These latter cells also divided (assuming that the G2/M length is sufficiently small compared to t_{M} – an assumption made to arrive at equation 8), but did not incorporate the BrdU label, since they had completed their S phase already before labelling started. Hence, to arrive at the approximation of the percentage of $\mathrm{BrdU}^{+}\mathrm{DNA}(2N)$ cells in equation 10, their contribution must be subtracted from the overall number of divided cells; it is given by twice the percentage of cells in G2M phase. In case that $c\ (t_{\mathrm{M}} -< T_{\mathrm{G2M}} >)\ <<\ 1$ does not hold, we can still use equation (7) together with $p_{\mathrm{G2M}} = e^{c\,<T_{\mathrm{G2M}}>} - 1$ to calculate cell cycle speed as a function of p_{G2M} and the percentage of $\mathrm{BrdU}^{+}\mathrm{DNA}(2N)$ cells.

It is important to note that no other cells born during the experiment will be missing in the $\mathrm{BrdU}^{+}\mathrm{DNA}(2N)$ gate because as long as the minimum S phase length is always greater than three hours, all of their parent cells were already in S phase when BrdU labeling began. For this reason, the expected loss of BrdU labeling efficiency does not affect the percentage of $\mathrm{BrdU}^{+}\mathrm{DNA}(2N)$ cells and we do not need to make any assumption about the pharmacokinetics of BrdU labeling.

For an exponential death time distribution, cell death cancels out in the steady state distributions $p^{\mathrm{ss}}(a)$ and $p_{\mathrm{tnd}}^{\mathrm{ss}}(\delta)$, as well as in the gate percentages. For more realistic distributions, (e.g., a log-normal death time distribution) we checked numerically that the effects on the steady state distributions are negligible and hence the gate percentages do not change considerably (Supplementary Figure 8c, d).

2. Quantification of the cell cycle phases: To estimate the mean durations of the G2M- and G1-phases, we used a short labelling duration, t_{M} = 0.5 h. At such a short labelling duration, cells that were in G1 or G2M at the time when BrdU was administered will be BrdU negative. There is no overlap with cells in S-phase since BrdU is fully available until the first half an hour and hence there are no unlabelled cells in S-phase. The fraction of cells in the gates that correspond to the G1- and G2M-phases are, respectively, given by

$$p\big(\mathrm{BrdU^{-}DNA}(2N)\big) = \int_0^{\infty} \mathrm{d}T_{\mathrm{G2M}}\, p^{\mathrm{ss}}(T_{\mathrm{G2M}}) \int_0^{\infty} \mathrm{d}T_{\mathrm{G1}}\, p^{\mathrm{ss}}(T_{\mathrm{G1}}) \int_{\max(0,t_{\mathrm{M}}-T_{\mathrm{G2M}})}^{T_{\mathrm{G1}}} \mathrm{d}a\, p^{\mathrm{ss}}(a), \quad (11)$$

$$p\big(\mathrm{BrdU^{-}DNA}(4N)\big) = \int_0^{\infty} \mathrm{d}T_{\mathrm{G2M}}\, p^{\mathrm{ss}}(T_{\mathrm{G2M}}) \int_0^{\max(T_{\mathrm{G2M}}-t_{\mathrm{M}},0)} \mathrm{d}\delta\, p_{\mathrm{tnd}}^{\mathrm{ss}}(\delta)\ . \quad (12)$$

A similar approximation scheme as discussed in Section 1 was applied to equations (11) and (12), leading to

$$p\big(\mathrm{BrdU^{-}DNA}(2N)\big) = 2\big(e^{-c\max((t_{\mathrm{M}}-<T_{\mathrm{G2M}}>),0)} - e^{-c<T_{\mathrm{G1}}>}\big), \quad (13)$$

$$p\big(\mathrm{BrdU^{-}DNA}(4N)\big) = e^{c\max((<T_{\mathrm{G2M}}>-t_{\mathrm{M}}),0)} - 1. \quad (14)$$

These equations, together with eq. (7) (evaluated for t_{M} = 3 h), were used to estimate the mean durations of the cell cycle phases together with the overall cell cycle length. The longer labelling duration of t_{M} = 3 h was chosen in order to i) have a sufficient number of $\mathrm{BrdU^{+}}$ daughter cells in the $\mathrm{BrdU^{+}DNA}(2N)$ gate, and ii) to ensure that many of the mother cells that have not divided yet, entered their S-phase after BrdU was not available anymore. The latter gives rise to a gap that naturally separates our daughter cells of interest from the mother cells that are in the beginning of their S-phase at time of the analysis (for a more detailed discussion see Section 3).

An advantage of this method is that it implicitly makes use of the limited BrdU availability (see Section 3 for more details), while it does not rely on any explicit information about the pharmacokinetics of BrdU labelling. In principle, the length of BrdU availability can be estimated by considering the equations for those gates that are characterized by a DNA content in between 2*N* and 4*N*; those gates explicitly depend on the time that BrdU is available to fully label cells.

It is important to note that although in the above approach two measurement time points are needed, our formalism could also be used to estimate the average cell cycle length based on

only one measurement time point. In this case, a late time point is chosen to evaluate eq. (10) and the fraction of cells in G2M is estimated based on the DNA content histograms using, for example, the Watson pragmatic curve fitting algorithm[4]. However, using a second time point increases the accuracy of the calculation of the total cell cycle length, and also allows for the estimation of the other cell cycle phases.

Our quantification procedure does not rely on the specific experimental setup combining BrdU labelling with DNA content quantification and can also be applied to similar DNA double labelling experiments (e.g., combining BrdU with the alternative thymidine analogue 5-Ethynyl-2´-deoxyuridine).

3. Possibility of undivided cells in the $\mathbf{BrdU^{+}DNA(2\mathit{N})}$ gate: To quantify the average speed with which cells progress through their cell cycle, our method focuses on cells that exit their S-phase and run through their G2M-phase during the experiment. For G1 durations that are on average comparable or bigger than the duration of the experiment, those cells are found in the $\mathrm{BrdU^{+}DNA}(2N)$ gate at the time of measurement. The formalism described in the previous sections implicitly assumes that this gate exclusively contains cells that divided between BrdU injection and DNA content quantification ("divided cells"). However, due to the limited sensitivity of DNA content measurements, the $\mathrm{BrdU^{+}DNA}(2N)$ gate can be occupied also by non-divided cells that have entered S-phase while BrdU was still available but have not synthesized enough DNA to have acquired a DNA content > 2N. This latter fraction of cells will be small if those cells, after BrdU became unavailable, had the time to sufficiently progress through S-phase such that they appear as DNA > 2N and no longer occupy the $\mathrm{BrdU^{+}DNA}(2N)$ gate. In other words, if BrdU is available for the duration t_{BrdU} and $\mathrm{t_M}$ is the measurement time, then a mother cell that has incorporated BrdU right before it becomes unavailable, has a time window of $t_{\mathrm{M}} - t_{\mathrm{BrdU}}$ to synthesize enough DNA content to leave the $\mathrm{BrdU^{+}DNA}(2N)$ gate; otherwise it will be misclassified in our formalism. The exact number of misclassified cells depends on the full distribution of S-phase lengths. In general, short S-phase lengths, short BrdU availabilities, and late measurement times are advantageous in this regard, as they enable more mother cells to leave the $\mathrm{BrdU^{+}DNA}(2N)$ gate in a given time interval. Thus, if the time point of analysis is chosen adequately, $\mathrm{BrdU^{+}}$ divided cells can be fully separated from $\mathrm{BrdU^{+}}$ non-divided cells according to their DNA content.

To investigate the BrdU availability and its link to the above-mentioned misclassification error, we generated synthetic BrdU/DNA data by means of stochastic simulations (see section 4 for more details). The simulation results indicate that indeed BrdU becomes unavailable long before the chosen measurement time of three hours. This can be inferred by the non-negligible

fraction of BrdU-unlabelled cells with DNA content between 2*N* and 4*N* in the data at 3 and 4.5 hours after BrdU administration—such cells would not exist in the case of continuous BrdU availability (Supplementary Figure 8a–b, middle and right panels). The comparison between the data and simulation suggests a BrdU availability window of around one hour, consistent with estimates in rodents[5].

4. Stochastic Simulation: In order to find the optimal time of measurement and to check the accuracy of our method, a stochastic simulation was implemented in MATLAB. For visualization, as shown in Supplementary Figure 8b and c, the *Flow Cytometry Graphical User Interface* was used. Mother cells were initialized at time zero with random inter-division times T_{Div}, drawn from a log-normal distribution $D(T_{\mathrm{Div}})$ and an age a drawn from $p^{\mathrm{ss}}(a)$. $p^{\mathrm{ss}}(a)$ itself is gained by simulating a tree of cells. After sufficiently many divisions, depending mainly on the variance of the underlying interdivision time distribution, steady state is reached and the age distribution can be gained numerically. The lengths of G2M- and S-phases were randomly drawn from corresponding log-normal distributions, rejecting values that would lead to $T_{\mathrm{G2M}} + T_{\mathrm{S}} > T_{\mathrm{Div}}$. Hence, the distributions of the individual cell cycle phases $D(T_i)$ drawn in this way are only approximately log-normal. The length of cell cycle phases for daughter cells were assigned similarly. BrdU availability was assumed to follow a Heaviside function $\Theta(t_{\mathrm{BrdU}} - t)$. A simulated cell was classified as BrdU positive, if its S-phase had a finite overlap with the BrdU availability interval. Gate percentages were obtained by considering a large number of mother cells. Finally, white Gaussian noise was added to the resulting DNA content and BrdU labelling intensity to reproduce BrdU/DNA plots that resemble the experimental data.

5. Robustness with respect to variability: Here we want to discuss, to which extent approximations like eq. (2) and (5) hold for our purposes. Using our stochastic simulation, we systematically showed that neglecting the variability in the length of the cell cycle and the individual phases is a justified assumption since it only results in small errors in the estimated quantities (Supplementary Figure 8e). To this end, we generated simulated data for which the BrdU availability and the full distributions of all cell cycle phases were known. We successively increased the coefficient of variation (CV) of the interdivision time distribution, thereby increasing the CVs of the individual phase distributions. Here we assumed for each phase that its contribution to the overall variance equals the contribution of its mean to the overall cell cycle length. Applying our method on these simulated data we back-calculated the mean of the overall interdivision time distribution as well as the means of the cell cycle phases. Relative errors of estimated parameters are shown in Supplementary Figure 8e. For this analysis, we made use of the estimated parameters for the CD62L^{+} cells of the control group; similar results

were obtained based on the parameters of the CD62L$^-$. We found that the estimation of the mean interdivision time was particularly robust with respect to variability in both cases.

Supplementary References

1 Buchholz, V. R. *et al.* Disparate individual fates compose robust CD8+ T cell immunity. *Science* **340**, 630-635, doi:10.1126/science.1235454 (2013).

2 Stukalin, E. B., Aifuwa, I., Kim, J. S., Wirtz, D. & Sun, S. X. Age-dependent stochastic models for understanding population fluctuations in continuously cultured cells. *J R Soc Interface* **10**, 20130325, doi:10.1098/rsif.2013.0325 (2013).

3 Dowling, M. R., Milutinovic, D. & Hodgkin, P. D. Modelling cell lifespan and proliferation: is likelihood to die or to divide independent of age? *J R Soc Interface* **2**, 517-526, doi:10.1098/rsif.2005.0069 (2005).

4 Watson, J. V., Chambers, S. H. & Smith, P. J. A pragmatic approach to the analysis of DNA histograms with a definable G1 peak. *Cytometry* **8**, 1-8, doi:10.1002/cyto.990080101 (1987).

5 Matiasova, A. *et al.* Flow cytometric determination of 5-bromo-2'-deoxyuridine pharmacokinetics in blood serum after intraperitoneal administration to rats and mice. *Histochem Cell Biol* **142**, 703-712, doi:10.1007/s00418-014-1253-7 (2014).
